# A Framework for Modeling Emerging Diseases to Inform Management

**DOI:** 10.3201/eid2301.161452

**Published:** 2017-01

**Authors:** Robin E. Russell, Rachel A. Katz, Katherine L.D. Richgels, Daniel P. Walsh, Evan H.C. Grant

**Affiliations:** U.S. Geological Survey, Madison, Wisconsin, USA (R.E. Russell, K.L.D. Richgels, D.P. Walsh);; University of Massachusetts, Amherst, Massachusetts, USA (R.A. Katz);; U.S. Geological Survey, Turner Falls, Massachusetts, USA (R.A. Katz, E.H.C. Grant);; University of Wisconsin, Madison (K.L.D. Richgels)

**Keywords:** zoonotic diseases, zoonoses, decision analysis, one health, emerging diseases, infectious diseases, mathematical modelling, model development, predictive modelling

## Abstract

The rapid emergence and reemergence of zoonotic diseases requires the ability to rapidly evaluate and implement optimal management decisions. Actions to control or mitigate the effects of emerging pathogens are commonly delayed because of uncertainty in the estimates and the predicted outcomes of the control tactics. The development of models that describe the best-known information regarding the disease system at the early stages of disease emergence is an essential step for optimal decision-making. Models can predict the potential effects of the pathogen, provide guidance for assessing the likelihood of success of different proposed management actions, quantify the uncertainty surrounding the choice of the optimal decision, and highlight critical areas for immediate research. We demonstrate how to develop models that can be used as a part of a decision-making framework to determine the likelihood of success of different management actions given current knowledge.

Despite continued calls to improve the response to emerging infectious zoonotic diseases (*1*,*2*), universal guidelines for determining the best course of action when a new disease emerges are unavailable. Increasing ease of global travel (*3*), continued encroachment of human populations into wildlife-occupied areas, climate change (*4*), and increasing rates of microbial evolution and antimicrobial drug resistance (*5*) have increased the likelihood that wildlife pathogens will be introduced into novel areas or naive populations and spill over into human populations (*1*). This accelerating rate of disease emergence leaves decision makers with a short time frame to determine and implement an appropriate course of action. A framework that quickly, rigorously, and effectively synthesizes relevant information about a wildlife pathogen in the early stages of emergence is essential for informing management at critical stages and ultimately reducing the potential effects of the disease on humans, livestock, and other wildlife populations.

Decision theoretic approaches provide formal guidelines for transparent, repeatable, and defensible decision-making that addresses specific management objectives, uncertainty of consequences, and potential trade-offs (*6*). Using approaches such as structured decision-making to frame decisions, modelers are provided a mechanism for including multiple and potentially competing objectives and evaluating the importance of uncertainties to a decision (*7*). An essential component for applying decision theory to emerging diseases is the development of predictive models that can be used to evaluate trade-offs between different management actions and disease consequences (*8*). The role of predictive models in informing management decisions is to estimate the consequences of alternative control strategies and help determine which strategies are optimal. Models can be used to assist decision makers with assessing the probability of a successful management outcome versus the risk of an unacceptable outcome (including nonecologic consequences), avoid unintentional consequences that might be exacerbated by delaying management interventions (*9*), and accommodate different goals and values of the decision maker and stakeholders (*5*,*8*). However, researchers are often reluctant to develop a model for forecasting the potential effects of emerging pathogens and the potential consequences of management actions because of uncertainty regarding the structure of the system (i.e., which parameters should be included in the model) and model parameter estimates (*10*).

Uncertainty often limits the ability to choose effective management strategies; therefore, it is vital to discriminate between uncertainties that are irreducible (i.e., environmental or demographic stochasticity, which might not be resolved with more information but must be considered regardless in making forecasts) and uncertainties that are reducible through research, monitoring, and surveillance. Reducible uncertainties might include the choice of model (i.e., structural uncertainty) that best describes system dynamics, the effects of system drivers (i.e., parametric uncertainty), and variation in system states across the landscape (i.e., spatial variability). Structural uncertainty can be resolved by testing different models and observing which model(s) best predict the system in future years. Parametric uncertainty and spatial variability can likewise be reduced with monitoring data or by conducting research.

In this article, we outline 3 components essential to building a predictive modeling framework that researchers and managers should consider early in the emergence of a wildlife pathogen: 1) which modeling frame is most appropriate, 2) which parameters or factors are critical to making preliminary predictions, and 3) how to collate existing data to parameterize the initial models. We describe 4 commonly used models for disease systems, identify 5 key characteristics of disease systems that represent minimally sufficient information needed to parameterize models, and identify 3 ways to parameterize models when reliable data are lacking. Using this 4-5-3 framework, researchers can work with managers to rapidly develop useful predictions with uncertainty and prioritize information gathering to improve the management of emerging diseases (Figure).

**Figure:**
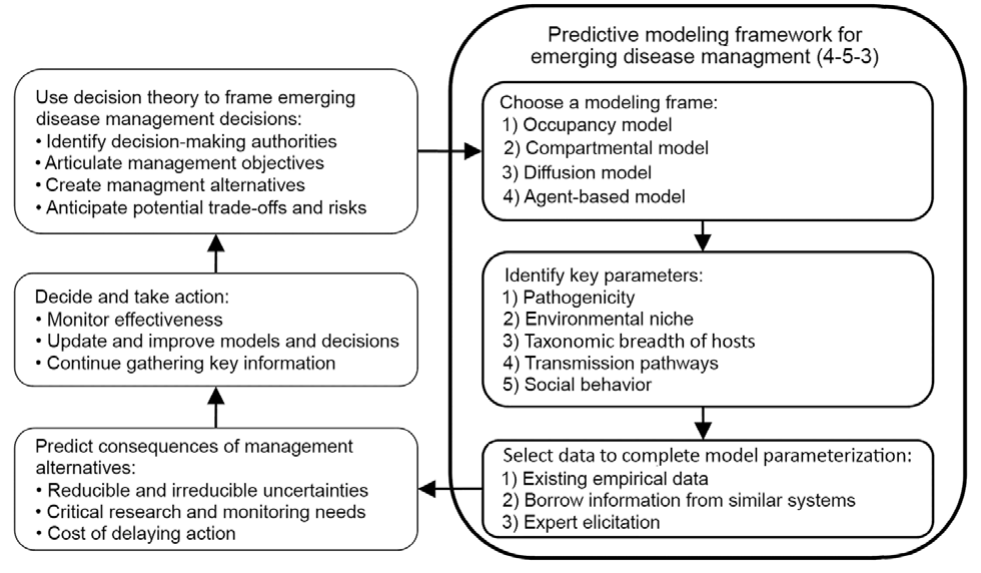
Predictive 4-5-3 modeling framework for emergency disease management.

## Choosing the Modeling Framework

Many disease modeling frameworks are available to select from (*11*,*12*) (Technical Appendix Table 1). By considering the objectives of the modeling, the assumptions of the different model frameworks, and the type of data that is either available or being collected, the list of modeling options can be narrowed down. Four generally useful classes of models are commonly used either on their own or in tandem with other model types to predict the spread and dynamics of wildlife pathogens: occupancy or patch dynamic models (*13*,*14*); compartmental (e.g., susceptible-infected-resistant) models (*15*); ecologic diffusion models (*16*); and agent-based (or individual-based) models (*17*).

Occupancy modeling focuses on patch dynamics, colonization, and extinction rates and is appropriate for hosts that live in discrete habitats, such as in wetlands, in forest or prairie remnants, or on mountain tops, where subpopulations are discrete and connected by occasional dispersal (*18*). The disease status (detected or not detected, percentage of hosts with disease) and the detection or nondetection of the host species in the patch is considered in these models, and the observed data can be corrected for nondetection bias. These models are appropriate for understanding landscape-level occurrence (number of patches occupied by disease) and extinction dynamics of an emerging disease (*19*). These models work best for disease systems in which the effects of the disease are severe and likely to result in patch extinction rather than sublethal effects that result in small declines in abundance. Alternatively, occupancy models have been used to model the dynamics of chytrid fungus for studies in which individual hosts within a patch are assessed for disease, and prevalence is estimated as the proportion of infected hosts (inferred via PCR detection of a pathogen) in a patch (*20*).

Compartmental models can capture the subtleties of sublethal effects on populations; these models require longitudinal information on individual hosts, although a sample of the population during 1 time period across multiple age groups can substitute for temporal information under certain assumptions (*21*). Traditional susceptible-infected-resistant models assume the population is homogeneous with little spatial structure. This type of model works well for host populations in which individual disease states can be observed through time (e.g., the host-disease system of brucellosis in bison, in which species are well-connected in space and can be captured and recaptured over time) (*22*).

Diffusion models can be used to model the spread of diseases and can be useful for predicting new areas of disease emergence. Information needed for these models includes host movement characteristics, contact rates between host species, and transmission pathways of the disease. Observations of new disease locations over time can also be used to estimate the rate of spread of the disease. Diffusion models have been used successfully to estimate the rates of spread of rabies in foxes (*23*) and foot-and-mouth disease in feral pigs (*24*).

Agent-based models (also known as individual-based models) can be used to assess the overall population dynamics of the host and the spread of the disease (*25*). These models can be particularly useful when it is necessary to model the disease system in a spatially-explicit fashion or when host behavior is complex (e.g., when hosts learn). Agent-based models have been used to assess the spatial patterns of parasite transmission in red colobus (*Procolobus rufomitratus*) monkeys, in which each host has a spatial memory of the value of patches, and each host weighs the benefits of being in a group for safety versus the costs of food competition (*25*). Only agent-based models are capable of capturing this complex behavior. By modeling what is known about individual host behavior and pathogen characteristics, systems-level patterns can be revealed by performing simulations. Agent-based models lend themselves to scenario development in which different patterns of host behavior can be modeled and the effects on the model outcome examined. These models, however, can be extremely data intensive, which impedes the modeling of systems with limited information (*25*,*26*).

 After selecting the framework among the different classes of models, model development usually progresses in a similar fashion. A common first step in model development is identifying the key characteristics of disease systems that are necessary to estimate the potential effects on the host population and identifying key points where management options will be most effective.

## Identifying Key Parameters

In general, 5 characteristics of a disease system are needed for predictive modeling: pathogenicity, environmental niche, taxonomic breadth of the hosts, transmission pathways between host and pathogen, and social behavior and movement patterns of the host species (Figure; Technical Appendix Table 2). Knowledge of each of these characteristics can be used in each of the 4 model frameworks, but the specific parameters used depends on the model chosen.

Pathogens can affect host species in a variety of ways, and management decisions should take into account the estimated long-term impacts on the population. Knowledge of the pathogenicity of the disease agent is essential for estimating long-term and population-scale effects. For example, diseases such as plague (*27*) might result in rapid die off of hosts, which might reduce the risk for pathogen spread beyond the local infected population. Some pathogens cause long-term sublethal effects, such as reduced fecundity or growth, and greater vulnerability to predators and other stressors (*28*), or they result in infected hosts that are long-lived and capable of infecting numerous other potential hosts (e.g., chronic wasting disease) (*29*).

The environmental niche of the disease agent or vector is also needed for developing models to predict the potential geographic extent of the disease (30). This information can help inform whether a disease might affect a species throughout its geographic range or whether environmental refuges might be expected (*31*). In addition, the taxonomic breadth of the hosts can indicate the potential for the pathogen to spread across multiple taxa, including humans. Multihost pathogens able to infect hosts across multiple taxonomic groups are more likely to cause emerging infectious diseases in humans or livestock (*32*).

Transmission pathways determine the rate at which the pathogen spreads and ultimately the spatial distribution of the disease (*33*). Knowledge of the transmission pathways is key to assessing the potential for the pathogen to have long-term and widespread effects, as well as evaluating the effectiveness of potential management actions. Mosquitoborne diseases, for example, have spread patterns very different from those for parasitic infections (e.g., toxoplasmosis, brain worm), which rely on specific hosts to complete their lifecycles; these differences lead to different predictions of spread (*34*,*35*).

Finally, the social behavior (which might be explicitly characterized by a contact network) of the host population can affect transmission rates by influencing the frequency and number of contacts (*36*–*38*). Panmictic populations (i.e., species that have interconnected populations mixing uniformly across their distribution) will be more likely to facilitate the rapid spread of disease compared with hosts that reside in small groups with low interpopulation connectivity. Similarly, hosts that commonly move long distances (such as bats or migratory birds) are more likely to facilitate rapid pathogen spread at large spatial scales. For example, the spread of white-nose syndrome among bats (https://www.whitenosesyndrome.org/resources/map) occurred over a relatively short period of time. Host species with large continuous spatial distributions (such as deer) also have an increased potential for spreading disease among populations on a continental scale, even when they might not individually travel long distances; however, their rate of geographic spread is generally slower (http://www.nwhc.usgs.gov/disease_information/chronic_wasting_disease/). Network theory has provided recent advances in the estimation and depiction of contact networks for disease transmission (*36*).

## Parameterization of the Model 

When little information is available regarding the true parameter estimates and variance, several options can be used for parameterization, including empirical observation (39), borrowing information from similar diseases (*40*), and expert elicitation (*41*). Typically, model parameterization will likely include a combination of sources and scientific experts depending on the emerging disease of interest and model frame selected.

Empirical observations of initial patterns and dynamics of pathogen spread can be used to estimate parameters, which can be updated as the pathogen is monitored through the initial introduction (*42*). Alternatively, observations from other areas where the pathogen previously emerged can be used to make initial predictions about introduction, spread, and establishment (*40*). Direct evidence of a disease agent’s potential for infection, transmission, and illness severity or death can be determined by laboratory trials and can identify which species might be most vulnerable to immediate population declines (*43*). Uncertainty primarily involves whether initial observations are characteristic of later infections on the basis of variations in disease processes and environmental conditions and whether ecologic niches are consistent among areas where the disease has and has not emerged.

A hallmark of emerging pathogens is that little empirical data exists, especially in the initial stages of emergence (*44*). The time required to obtain empirical data on a disease agent might be costly in terms of windows for effective action and should be explicitly evaluated in initial research efforts. However, borrowing information from more thoroughly described pathogens that cause similar diseases and expert elicitation might include additional uncertainty that can only be resolved through observation of the disease of interest. Despite these uncertainties, delaying management actions while information is collected might reduce effectiveness of the management strategy, limit available actions, and result in unacceptable population declines. Instead of waiting for results from empirical studies, information from other related diseases can be used for parameterization of a novel disease model. This borrowing-of-information method used to estimate parameters can include both the uncertainty in the estimates from the original disease (i.e., variance), and the uncertainty in the relatedness between the novel and the original pathogen (which can be deduced by phylogenetic distances, origin, or environmental niche differences, if these are known or can be estimated).

In combination with empirical observation and borrowed information, modelers can use expert elicitation methods to formally query experts for parameter estimates (online Technical Appendix) (*45*). A variety of methods exist to reduce biases associated with acquiring subjective information from experts, but all of these methods involve identifying explicitly the parameters for which expert opinion is needed; preparing experts to normalize beliefs and experience (e.g., providing experts with common literature and explaining to them the uncertain parameters); summarizing and discussing the rationale; and quantifying individual and group uncertainty. A strength of expert elicitation during early stages of disease emergence is that it permits rapid evaluation of management alternatives (e.g., control, eradication) under system and parameter uncertainty.

## Uncertainty

After initial parameterization of a given model, an analysis of the sensitivity and uncertainty associated with the model should be conducted. In general, sensitivity analyses examine the contribution of each predictor variable to the uncertainty in the response variable, while uncertainty analyses describe the examination of the range of outcomes possible given the uncertainty in the input variables (*46*). Multiple methods are available for assessing the extent of the uncertainty associated with various parameters, including variance-based methods, global uncertainty and sensitivity analyses, and Bayesian belief networks, which can help identify the uncertainties that are most likely to affect the management decision (*47*). These uncertainties can then become the focus of future research and monitoring efforts (*48*,*49*). Decision models that can evaluate trade-offs among multiple objectives (such as multicriteria decision analysis and portfolio decision analysis) (*49*) under uncertainty and evaluate different optimal policies over time (stochastic dynamic programming and Markov decision process models) can be integrated with probabilistic disease predictive models to provide insights about optimal disease management strategies under deep uncertainty.

## Conclusions

Identifying robust management strategies in the early stages of disease emergence, when more control options are available, is limited by numerous uncertainties. Predictive models can be useful in evaluating control options, forecasting spread, and calculating risk (the potential for an outcome to occur and the uncertainty surrounding the outcome), but parameterization of such models for emerging wildlife diseases is challenging. By outlining 4 common models, 5 key parameters, and 3 methods for obtaining data, we outline a process for developing useful predictive models within a decision analysis framework (Figure). Ultimately, the development of models that capture key aspects of pathogen transmission and the severity of its effects can be used to evaluate the utility of different management decisions, to determine where to focus limited resources, and to identify and justify immediate research needs (*50*). As a burgeoning human population continues to encroach on wildlife habitats, encounters between humans and wildlife will likely become more common. Identifying diseases that have the potential to profoundly impact human, livestock, and ecosystem health, and responding in a rapid and logical manner is a priority. Control and mitigation of emerging diseases will benefit from the early development and application of predictive modeling frameworks.

**Table:** Key information needs for management of emerging diseases of wildlife

Pathogen characteristics	Description
Pathogenicity	What is the severity, lethality, and rapidity (rate of mortality) of effects on hosts?
Environmental niche	What environmental conditions (temperature, humidity) restrict persistence?
Taxonomic breadth of host	Is there evidence that the agent type can affect hosts across multiple taxa? Which taxa?
Host characteristics	
Contact networks	Spatial structure: What is the spatial structure of host populations: panmictic, metapopulations, or isolated? Does this vary across the landscape?
	Movement patterns: What is the average and maximum distance an infected host might travel?
	Social behavior: What is the social behavior of individual members in the host populations? What is the rate of contact between species?
Transmission pathways	How is the pathogen transmitted between hosts? How many different transmission pathways are there?

Technical Appendix Examples demonstrating the 4 model types, the 5 key parameters, and the 3 methods for completing parameterization of models when insufficient evidence exists.
