# Supplementary material for: A Framework for Modeling Emerging Diseases to Inform Management
Source: Emerg Infect Dis. 2017 Jan;23(1):1–6. doi: 10.3201/eid2301.161452 (PMC5176225; doi:10.3201/eid2301.161452)
Supplement: Technical Appendix — Examples demonstrating the 4 model types, the 5 key parameters, and the 3 methods for completing parameterization of models when insufficient evidence exists. [file 16-1452-Techapp-s1.pdf]

Article DOI: http://dx.doi.org/10.3201/eid2301.161452

A Framework for Modeling Emerging Diseases to Inform Management

Technical Appendix

Application of framework to hypothetical scenario.

Scenario: Salamander chytridiomycosis, caused by a fungal pathogen, *Batrachochytrium salamandrivorans*, is detected from an unusual die-off occurrence in a U.S. Fish and Wildlife Refuge Complex in the Mid-Atlantic region of the United States. Park staff and visitors detected several dead and dying newts near a pond that is a popular fishing location. Currently, only one pond has been identified as containing infected eastern newts (*Notophthalmus viridescens*). At this time, a dozen specimens have been collected and all have tested positive for the fungus. The true population size of newts in the pond is unknown. There are at least three other potential breeding areas within 1 km of the infected pond.

Pathogenicity: The fungus has been shown to cause high rates of mortality in susceptible salamander species and in particular newts (online Technical Appendix Table 3) (*1*).

Environmental niche: The temperature and humidity niche of *B. salamandrivorans* is predicted to include large areas of the Mid-Atlantic (*2*,*3*).

Taxonomic breadth of hosts: Most fungal diseases are taxa specific; therefore, the risk for spread of the disease to other taxa (mammals or birds) is likely low (*4*).

Transmission pathway: Direct contact has been shown to be an effective transmission pathway for the fungus (*1*), but indirect transmission is possible with fungal spores potentially being carried by water, on fomites, and by other organisms (*4*).

Social behavior/connectivity: In general, eastern newts live in metapopulations (i.e., small populations connected to the larger population through occasional dispersal events) (*5*). Gill et al. identified two movement periods in eastern newts: a movement to breeding ponds in spring and migration periods to terrestrial habitat in the fall (*5*). Though the literature on newt movements is sparse, Regosin et al. determined that fewer than 20% of the population traveled

more than 100 m from breeding ponds (*6*). However, Roe and Grayson found that efts (i.e., juvenile stages of newts) moved up to 50 m a night indicating the potential for longer distance movements (*7*). Finally, contact during breeding season (March–August) is increased for species with aggregate breeding behaviors (e.g., spotted salamanders) but may be reduced during other times of the year.

Technical Appendix Table 1. Examples from the literature describing the use of different modeling frameworks to describe disease processes

Model type	Examples	Software
Occupancy	chytrid fungus in amphibians (*8*)<br>flea species presence on prairie dogs (*9*)<br>parasites in birds (*10*)<br>chytrid fungus in amphibians (*11*)<br>disease dynamics in metapopulations (*12*)<br>whirling disease in fish (*13*)<br>insects and palm trees (*14*)	Unmarked: https://cran.r-project.org/web/packages/unmarked/index.html<br>Presence: http://www.mbr-pwrc.usgs.gov/software/presence.html<br>MARK: http://warnercnr.colostate.edu/~gwhite/mark/mark.htm
Compartmental	brucellosis in bison (*15*)<br>chronic wasting disease in deer (*16*)<br>macroparasites and wildlife (*17*)<br>bacterial infections and possums (*18*)<br>harvest effects on wildlife disease (*19*)<br>West Nile virus and birds (*20*)<br>control strategies and swine flu (*21*)<br>dengue and mosquitoes (*22*)	EpiModel: http://www.epimodel.org/<br>Outbreak Tools: https://cran.r-project.org/web/packages/OutbreakTools/index.html<br>simecol:https://cran.r-project.org/web/packages/simecol/index.html<br>Any ordinary differential equation solver: Matlab, Simulink, Excel, deSolve in R
Diffusion	rabies in foxes (*23*)<br>foot and mouth disease in pigs (*24*)<br>rabies in raccoons (*25*)<br>pathogens and bumblebees (*26*)<br>plague traveling waves (*27*)<br>chronic wasting disease (*28*)	spate: https://cran.r-project.org/web/packages/spate/index.html<br>simecol: https://cran.r-project.org/web/packages/simecol/index.html
Agent-based	parasite transmission in monkeys (*29*)<br>rabies in foxes (*30*)<br>swine fever in wild boar (*31*)<br>territoriality and sociality of wolves (*32*)<br>foot and mouth outbreak in cattle (*33*)	NetLogo: http://ccl.northwestern.edu/netlogo/ or RNetlogo: http://rnetlogo.r-forge.r-project.org/<br>Ecolab: http://ecolab.sourceforge.net/<br>Flame: http://www.flame.ac.uk/<br>Pandora: http://www.bsc.es/computer-applications/pandora-hpc-agent-based-modelling-framework<br>Simecol: https://cran.r-project.org/web/packages/simecol/index.html

Technical Appendix Table 2. Detailed description of parameter estimates associated with each characteristic, and techniques associated with the estimation of those parameters

Key parameters	Specific parameters	Techniques
Pathogenicity	Survival and reproduction of diseased and nondiseased hosts<br>Occupancy of patches<br>Population density in areas with and without disease	Mark-recapture of diseased and uninfected hosts<br>Visual counts of diseased and uninfected hosts<br>Presence/nondetection of hosts in areas with and without disease
Environmental niche	Relationship of pathogen growth, virulence, and survival with environmental conditions such as temperature and humidity	Laboratory studies to determine pathogen response to environmental conditions.<br>Presence/nondetection of pathogen in field surveys: environmental DNA
Taxonomic breadth of hosts	Pathogenicity for multiple hosts	Challenge experiments in the laboratory<br>Field surveys to detect exposure or pathogen in multiple hosts<br>Passive or active surveillance for pathogen presence, exposure, and related morbidity
Transmission pathways	Rate of transmission (if any) host to host, environment to host, vector to host, etc.<br>Rates at which contacts result in host to host disease transmission	Laboratory experiments fulfilling Koch's postulates<br>Field surveys to identify reservoirs and vectors<br>Models to replicate observed dynamics<br>Genetic studies of the pathogen<br>Comparative genetics of potential hosts and vectors to pathogens

Key parameters	Specific parameters	Techniques
Social behavior/contact networks	Contact rates: rates at which individual species contact members of their own social group and other social groups, rates at which infectious animals move	Identification of host genetic structure or nonlethal microbes in hosts<br>Mark recapture: spatially-explicit and multistate<br>Telemetry<br>Proximity collars<br>Observational studies<br>Spatial patterns of nonlethal microbes in hosts

Technical Appendix Table 3. Identification of key parameters for *Batrachochytrium salamandrivorans*, an emerging disease of salamanders*

Key parameters	Description	Estimates
Pathogenicity<br>Environmental niche	Disease is highly pathogenic for susceptible species (*1*).<br>Current models indicate conditions for *B. salamandrivorans* exist throughout the Eastern seaboard (*2*,*3*).	Mortality 0.90–1.00<br>Probability environmental niche includes local populations = 1<br>Probability niche includes all of species range 0.90–1.00
Taxonomic breadth of hosts	Current information indicates the probability is low that species outside of salamanders are affected.	1% chance of infecting other taxa
Social behavior/connectivity	Spatial structure: salamanders in this area live in small isolated ponds (metapopulations).<br>Movement patterns: salamander mobility is relatively limited (unlikely to travel more than a few hundred meters). Juveniles are the dispersing class and may travel farther.<br>Social behavior: salamanders in ponds live in close proximity to one another during the breeding season.	Probability of contact with infected host within the same pond is high (0.75–1.00).<br>Probability of contact with an infected host from another pond is likely a function of distance.<br>Ponds connected by riparian areas facilitate movements.
Transmission pathways	There is much uncertainty surrounding transmission pathways. Direct contact can pass fungal spores between hosts, it is also possible that fungal spores can be passed from an individual to the environment or other organisms and then to other individuals. Possibly spores can be carried on fomites.	Probability host-to-host contact leads to an infection is high (0.80–1.00).<br>Probability host infection can occur from environment is low (0–0.10).

*Ideally parameter estimates would represent the best guess estimates of multiple experts.

References

1. Martel A, Blooi M, Adriaensen C, Van Rooij P, Beukema W, Fisher MC, et al. Recent introduction of a chytrid fungus endangers Western Palearctic salamanders. Science. 2014;346:630–1. PubMed http://dx.doi.org/10.1126/science.1258268

2. Yap TA, Koo MS, Ambrose RF, Wake DB, Vredenburg VT. Biodiversity. Averting a North American biodiversity crisis. Science. 2015;349:481–2. PubMed http://dx.doi.org/10.1126/science.aab1052

3. Richgels KE, Russell RE, Adams MJ, White CL, Campbell Grant EHC. Spatial variation in risk and consequence of *Batrachochytrium salamandrivorans* introduction in the USA. R Soc Open Sci. 2016;3:150616. PubMed http://dx.doi.org/10.1098/rsos.150616

4. Fisher MC, Henk DA, Briggs CJ, Brownstein JS, Madoff LC, McCraw SL, et al. Emerging fungal threats to animal, plant, and ecosystem health. Nature. 2012;484:186–94. PubMed http://dx.doi.org/10.1038/nature10947

5. Gill DE. The metapopulation ecology of the red-spotted newt, *Notophthalmus viridescens* (Rafinesque). Ecol Monogr. 1978;48:145–66. http://dx.doi.org/10.2307/2937297

6. Regosin JV, Windmiller BS, Homan RN, Reed JM. Variation in terrestrial habitat use by four pool-breeding amphibian species. J Wildl Manage. 2005;69:1481–93. http://dx.doi.org/10.2193/0022-541X(2005)69[1481:VITHUB]2.0.CO;2

7. Roe AW, Grayson KL. Terrestrial movements and habitat use of juvenile and emigrating adult eastern red-spotted newts, *Notophthalmus viridescens*. J Herpetol. 2008;42:22–30. http://dx.doi.org/10.1670/07-040.1

8. Miller DAW, Talley BL, Lips KR, Campbell Grant EH. Estimating patterns and drivers of infection prevalence and intensity when detection is imperfect and sampling error occurs. Methods Ecol Evol. 2012;3:850–9. http://dx.doi.org/10.1111/j.2041-210X.2012.00216.x

9. Eads DA, Biggins DE, Doherty PF Jr, Gage KL, Huyvaert KP, Long DH, et al. Using occupancy models to investigate the prevalence of ectoparasitic vectors on hosts: an example with fleas on prairie dogs. Int J Parasitol Parasites Wildl. 2013;2:246–56. PubMed http://dx.doi.org/10.1016/j.ijppaw.2013.09.002

10. Lachish S, Gopalaswamy AM, Knowles SCL, Sheldon BC. Site-occupancy modelling as a novel framework for assessing test sensitivity and estimating wildlife disease prevalence from imperfect diagnostic tests. Methods Ecol Evol. 2012;3:339–48. http://dx.doi.org/10.1111/j.2041-210X.2011.00156.x

11. Schmidt BR, Kery M, Ursenbacher S, Hyman OJ, Collins JP. Site occupancy models in the analysis of environmental DNA presence/absence surveys: a case study of an emerging amphibian pathogen. Methods Ecol Evol. 2013;4:646–53. http://dx.doi.org/10.1111/2041-210X.12052

12. Gog J, Woodroffe R, Swinton J. Disease in endangered metapopulations: the importance of alternative hosts. Proc Biol Sci. 2002;269:671–6. PubMed http://dx.doi.org/10.1098/rspb.2001.1667

13. Thompson KG. Use of site occupancy models to estimate prevalence of *Myxobolus cerebralis* infection in trout. J Aquat Anim Health. 2007;19:8–13. PubMed http://dx.doi.org/10.1577/H06-016.1

14. Abad-Franch F, Ferraz G, Campos C, Palomeque FS, Grijalva MJ, Aguilar HM, et al. Modeling disease vector occurrence when detection is imperfect: infestation of Amazonian palm trees by triatomine bugs at three spatial scales. PLoS Negl Trop Dis. 2010;4:e620. PubMed http://dx.doi.org/10.1371/journal.pntd.0000620

15. Dobson A, Meagher M. The population dynamics of brucellosis in the Yellowstone National Park. Ecology. 1996;77:1026–36. http://dx.doi.org/10.2307/2265573

16. Miller MW, Hobbs NT, Tavener SJ. Dynamics of prion disease transmission in mule deer. Ecol Appl. 2006;16:2208–14. PubMed http://dx.doi.org/10.1890/1051-0761(2006)016[2208:DOPDTI]2.0.CO;2

17. Heesterbeek JAP, Roberts MG. Mathematical models for microparasites of wildlife. In: Grenfell BT, Dobson AP, editors. Ecology of infectious diseases in natural populations. Cambridge: Cambridge University Press. 1995. p. 90–122.

18. Caley P, Ramsey D. Estimating disease transmission in wildlife, with emphasis on leptospirosis and bovine tuberculosis in possums, and effects of fertility control. J Appl Ecol. 2001;38:1362–70. http://dx.doi.org/10.1046/j.0021-8901.2001.00676.x

19. Choisy M, Rohani P. Harvesting can increase severity of wildlife disease epidemics. Proc Biol Sci. 2006;273:2025–34. PubMed http://dx.doi.org/10.1098/rspb.2006.3554

20. Wonham MJ, Lewis MA, Rencławowicz J, van den Driessche P. Transmission assumptions generate conflicting predictions in host-vector disease models: a case study in West Nile virus. Ecol Lett. 2006;9:706–25. PubMed http://dx.doi.org/10.1111/j.1461-0248.2006.00912.x

21. Bolzoni L, Real L, De Leo G. Transmission heterogeneity and control strategies for infectious disease emergence. PLoS One. 2007;2:e747. PubMed http://dx.doi.org/10.1371/journal.pone.0000747

22. Erickson RA, Presley SM, Allen LJS, Long KR, Cox SB. A dengue model with a dynamic *Aedes albopictus* vector population. Ecol Modell. 2010;221:2899–908. http://dx.doi.org/10.1016/j.ecolmodel.2010.08.036

23. Källén A, Arcuri P, Murray JD. A simple model for the spatial spread and control of rabies. J Theor Biol. 1985;116:377–93. PubMed http://dx.doi.org/10.1016/S0022-5193(85)80276-9

24. Pech RP, McIlroy JC. A model of the velocity of advance of foot and mouth disease in feral pigs. J Appl Ecol. 1990;27:635–50. http://dx.doi.org/10.2307/2404308

25. Moore DA. Spatial diffusion of raccoon rabies in Pennsylvania, USA. Prev Vet Med. 1999;40:19–32. PubMed http://dx.doi.org/10.1016/S0167-5877(99)00005-7

26. Otterstatter MC, Thomson JD. Does pathogen spillover from commercially reared bumble bees threaten wild pollinators? PLoS One. 2008;3:e2771. PubMed http://dx.doi.org/10.1371/journal.pone.0002771

27. Adjemian JZ, Foley P, Gage KL, Foley JE. Initiation and spread of traveling waves of plague, *Yersinia pestis*, in the western United States. Am J Trop Med Hyg. 2007;76:365–75. PubMed

28. Garlick MJ, Powell JA, Hooten MB, McFarlane LR. Homogenization of large-scale movement models in ecology. Bull Math Biol. 2011;73:2088–108. PubMed http://dx.doi.org/10.1007/s11538-010-9612-6

29. Bonnell TR, Sengupta RR, Chapman CA, Goldberg TL. An agent-based model of red colobus resources and disease dynamics implicates key resource sites as hot spots of disease transmission. Ecol Modell. 2010;221:2491–500. http://dx.doi.org/10.1016/j.ecolmodel.2010.07.020

30. Jeltsch F, Müller MS, Grimm V, Wissel C, Brandl R. Pattern formation triggered by rare events: lessons from the spread of rabies. Proc Biol Sci. 1997;264:495–503.http://dx.doi.org/10.1098/rspb.1997.0071 PubMed

31. Kramer-Schadt S, Fernández N, Eisinger D, Grimm V, Thulke H-H. Individual variations in infectiousness explain long-term disease persistence in wildlife populations. Oikos. 2009;118:199–208. http://dx.doi.org/10.1111/j.1600-0706.2008.16582.x

32. Pitt WC, Box PW, Knowlton FF. An individual-based model of canid populations: modelling territoriality and social structure. Ecol Modell. 2003;166:109–21. http://dx.doi.org/10.1016/S0304-3800(03)00130-3

33. Keeling MJ, Woolhouse MEJ, Shaw DJ, Matthews L, Chase-Topping M, Haydon DT, et al. Dynamics of the 2001 UK foot and mouth epidemic: stochastic dispersal in a heterogeneous landscape. Science. 2001;294:813–7. PubMed http://dx.doi.org/10.1126/science.1065973
